# Genomic analysis of urogenital and rectal *Neisseria meningitidis* isolates reveals encapsulated hyperinvasive meningococci and coincident multidrug-resistant gonococci

**DOI:** 10.1136/sextrans-2016-052781

**Published:** 2017-01-30

**Authors:** Odile B Harrison, Kevin Cole, Joanna Peters, Fiona Cresswell, Gillian Dean, David W Eyre, John Paul, Martin CJ Maiden

**Affiliations:** 1 Department of Zoology, University of Oxford, Oxford, UK; 2 Brighton and Sussex University Hospitals NHS Trust, Brighton, UK; 3 Nuffield Department of Medicine, University of Oxford, Oxford, UK

**Keywords:** NEISSERIA GONORRHOEA, NEISSERIA MENINGITIS, INFECTIOUS DISEASES, MENINGITIS

## Abstract

**Objective:**

Invasive meningococcal disease (IMD) outbreaks in men who have sex with men (MSM) have been associated with meningococcal colonisation of the urethra and rectum, but little is known about this colonisation or co-colonisation with the closely related gonococcus. Whole genome sequencing (WGS) was employed to explore these phenomena.

**Methods:**

Meningococci isolated from the urogenital tract and rectum (n=23) and coincident gonococci (n=14) were analysed by WGS along with contemporary meningococci from IMD (n=11). All isolates were obtained from hospital admissions in Brighton, UK, 2011–2013. Assembled WGS were deposited in the PubMLST/neisseria database (http://pubmlst.org/neisseria) and compared at genomic loci common to gonococci or meningococci.

**Results:**

As expected, most meningococci from IMD were encapsulated and belonged to hyperinvasive lineages. So too were meningococci found in the urogenital tract and rectum, contrasting to those asymptomatically carried in the nasopharynx where such meningococci are rare. Five hyperinvasive meningococcal lineages and four distinct gonococcal genotypes were recovered, including multiresistant ST-1901 (NG MAST-1407) gonococci.

**Conclusions:**

These data were consistent with a predisposition for potentially virulent encapsulated hyperinvasive meningococci to colonise the urethra and rectum, which suggests their involvement in MSM IMD outbreaks. The coincidence of multiresistant gonococci raises wider public health concerns.

## Introduction

*Neisseria meningitidis* and *Neisseria gonorrhoeae* are two closely related Gram-negative diplococcal bacteria responsible for invasive meningococcal disease (IMD) and gonorrhoea, respectively. Their genetic similarity indicates they diverged from the same ancestral population in the relatively recent past.^1^ Given the much lower diversity of the gonococcus, it is thought to have evolved by a change of niche from the nasopharynx, where most *Neisseria* are found, to the urogenital tract, but paradoxically, reports of clinically recognisable gonococcal disease are much older (1376, and perhaps much earlier)^2^ than meningococcal disease, which is widely considered to have been first recognised in 1805.^3^

Meningococcal disease can be thought of as an emerging infectious disease of the early 1800s, occurring in a growing human population, which, at the beginning of the 19th century, had exceeded 1 billion.^4^ IMD is predominantly caused by meningococci expressing a polysaccharide capsule, the biochemical and genetic composition of which determine the serogroup, with six serogroups (A, B, C, W, X and Y) causing almost all IMD.^5^ In addition, most invasive meningococci belong to fewer than 20 genotypes, known as ‘hyperinvasive lineages’, which are identified, by multilocus sequence typing (MLST), as groups of related sequence types (STs) called clonal complexes (ccs).^6^ ^7^ Almost all infections caused by meningococci result from asymptomatic carriage and IMD is inimical to spread as it does not result in transmission.

While the human nasopharynx remains the site that is preferentially colonised by *N. meningitidis*, an increasing number of urogenital and rectal meningococcal infections have been reported, accompanied by evidence of transmission between sexual partners involving the nasopharynx, urogenital tract and rectum.^8^ ^9^ These transmission pathways may have facilitated the IMD outbreaks recently reported among men who have sex with men (MSM) in Canada, the United States, Germany, France and Belgium.^10–13^ These outbreaks were associated with serogroup C meningococci belonging to the hyperinvasive ST-11 clonal complex, prompting the use of vaccination with the quadrivalent ACWY conjugate vaccine as a public health response.^14^ Although these outbreaks indicate that urogenital and rectal colonisation by *N. meningitidis* may increase opportunities for transmission and infection, little is known about the prevalence and strain characteristics of meningococci in the urogenital tract and rectum. Here, we present genomic analyses and comparison of such organisms from Brighton, UK, which were temporally matched with whole genome sequencing (WGS) data obtained from cases of IMD from the same city. The increasing presence of meningococci in the urogenital tract may represent a niche into which this organism is expanding, perhaps leading to the emergence of novel infectious variants that mirror the emergence of gonococcal disease and this warrants surveillance.

## Materials and methods

### Clinical setting and samples

Isolates originated from routine diagnostic samples submitted to microbiology at the Brighton and Sussex University Hospitals NHS Trust (BSUH) Brighton, UK, calendar years 2011–2013. *N. meningitidis* isolates were obtained from acute medical patients with invasive disease and also from patients attending sexual health services (c25000 attendances/year, 25% MSM) in whom *N. meningitidis* was isolated incidentally during routine urethral and rectal screening for gonorrhoea. During the study period, 11 meningococci were obtained and archived from cases of invasive disease (7 blood culture, 2 synovial fluid, 2 cerebrospinal fluid): information on the sexual orientation was not available. During the same period, 36 urogenital/rectal meningococci were detected, of which 7/36 were urethral and 29/36 were rectal samples: 23/36 viable urogenital/rectal *N. meningitidis* isolates were available for further study. As pharyngeal meningococci were not considered clinically significant, they were not routinely archived. Between 2011 and 2013, a total of 1033 *N. gonorrhoeae* isolates were recorded with 12 patients (all male) yielding both *N. meningitidis* and *N. gonorrhoeae* (5 concurrently, 7 more than a month apart). Fourteen gonococci from these patients were available for study.

*N. gonorrhoeae* isolates were initially identified using a nucleic acid amplification test (BD ProbeTec, BD, Franklin Lakes, New Jersey, USA).^15^ *N. gonorrhoeae* genomic DNA was extracted from isolates subcultured onto VCAT (Vancomycin, Colistin sulfate, Amphotericin B, Trimethoprim) selective agar (Oxoid, Basingstoke, UK) and incubated in a CO_2_-rich atmosphere at 37°C for 24 hours. Prior to DNA extraction, *N. meningitidis* isolates were streak plated onto Columbia agar plus 5% (v/v) sheep blood and incubated overnight at 37°C in an atmosphere containing 5% CO_2_. Gonococci and meningococci were stored in 20% glycerol broth at −80°C. Antimicrobial susceptibilities were determined using agar diffusion with Etest and published British Society for Antimicrobial Chemotherapy (BSAC) guidelines for minimum inhibitory concentration (MIC) interpretation.^16^

### WGS and analysis

Genomic gonococcal DNA was extracted using a commercial kit (QuickGene, Fujifilm, Tokyo, Japan). Meningococcal genomic DNA was extracted using the Wizard Genomic DNA purification kit (Promega). Both meningococci and gonococci were sequenced using the Illumina HiSeq platform and short-read sequences were assembled de novo using the VelvetOptimiser assembly program. Resultant assemblies were uploaded to the pubMLST database running the BIGSdb genomic platform hosted on http://www.pubMLST.org/neisseria. WGS data from a French cc11 serogroup C meningococcus (LNP27256), originating from a French MSM outbreak, were also included in the analyses as well as the genome from a cc4821 *N. meningitidis* Chinese isolate, 053442.^12^ ^17^

PubMLST.org/neisseria archives and annotates, at the time of writing, >7000 WGS data from across the *Neisseria* genus. WGS deposited in the database are automatically annotated for any defined loci, identifying alleles ≥98% sequence identity and updating isolate records with allele numbers. This enabled the genogroup, PorA and FetA type as well as MLST ST or NG MAST to be identified. Chromosomal and plasmid genes in addition to intergenic regions implicated in gonococcal antimicrobial resistance (AMR) have also been defined in the pubMLST *Neisseria* database with alleles containing known mutations associated with AMR annotated accordingly.^18^ ^19^

The BIGsdb Genome comparator tool, implemented within the website, was employed to compare WGS data.^20^ Using this tool, 1605 loci identified as core to meningococci and belonging to the *N. meningitidis* core genome MLST (cgMLST) scheme V.1.0 were compared between all meningococci.^21^ In addition, 1668 loci, identified as core to gonococci in this dataset and defined in the *N. gonorrhoeae* cgMLST scheme V.1.0, were compared between all gonococci. Using the genome comparator tool, coding sequences from selected loci are extracted and compared against assembled WGS data. Alleles at each locus are designated with an integer, identifying isolates with the same or different allelic profiles, and a distance matrix is generated based on the number of variable alleles resolving isolates into networks using the NEIGHBORNET algorithm and a standalone instance of SPLITSTREE.^22^ ^23^ Gene-by-gene comparisons undertaken using genome comparator also provide lists of loci identical, variable, missing or incomplete between datasets thereby resolving population relationships and elucidating where genetic variation is taking place. This in turn allows the functional significance of such variation to be identified.

## Results

### Isolate characterisation

During the study period, *N. meningitidis* was obtained more frequently from rectal (19/23, 83%) than urethral samples (4/23, 17%) but was detected less frequently than *N. gonorrhoeae* (365/1033, 32% rectal; 435/1033, 47% urethral; 185/1033, 16% pharyngeal; 45/1033, 4% endocervix; 3/1033, 0.2% other sites including conjunctiva and intrauterine contraceptive device). Thirteen (56%) of the 23 urogenital *N. meningitidis* isolates belonged to clonal complexes associated with IMD (table 1). There were also clonal complexes more commonly found in carriage, for example, cc1157 (n=3), and isolates with STs not currently associated with a clonal complex (table 1). Isolates from clonal complexes not associated with IMD were either unencapsulated, possessing the capsule null locus (*cnl)* or were serogroups E or Z. Most of the remaining isolates were serogroup B (67%) belonging to clonal complexes cc41/44, cc269, cc4821 as well as the ST-1976 isolates, while the cc11 isolates were serogroup C and the cc23 isolates were serogroup Y. There were three cc1157 isolates and these were serogroup B, E or Z (table 1).

**Table 1:** Isolate collection

Isolate	Source	Concomitant gonococcal infection	Sex	Strain type (fine type)	Accession number
Urogenital *Neisseria meningitidis* isolates					
cc11					
NM8633	Rectum	No	M	C: P1.5-1,10-8: F3-6: ST-11	ERR585992
cc23					
NM10762*	Rectum	No	M	Y: P1.5-1,10-1: F4-1: ST-1655	ERR586012
cc41/44					
NM8736	Rectum	J10	M	B: P1.17-1,23: F1-5: ST-1097	ERR585996
NM9853	Urethra	No	M	B: P1.7-2,4: F1-5: ST-41	ERR586003
NM10833	Rectum	No	M	B: P1.5-2,10-1: F5-9: ST-10867	ERR586014
cc269					
NM8525*	Urethra	No	M	B: P1.19-1,15-11: F5-1: ST-269	ERR585987
NM8468	Rectum	No	M	B: P1.19,15-1: F1-5: ST-10864	ERR585986
NM8583	Rectum	No	M	B: P1.19,15-1: F1-5: ST-10864	ERR585990
NM8726	Rectum	No	M	B: P1.19,15-1: F1-5: ST-10864	ERR585995
cc1157					
NM8572	Rectum	No	M	B: P1.7-12,14: F1-7: ST-10865	ERR585989
NM10421	Rectum	K11	M	E: P1.17,9: F1-15: ST-3203	ERR586010
NM10989	Rectum	No	M	Z: P1.21-7,16: F5-36: ST-1157	ERR586018
cc4821					
NM8652	Rectum	No	M	B: P1.20,23: F3-36: ST-3200	ERR585993
NM9658	Rectum	A1	M	B: P1.20,23-2: F3-36: ST-3200	ERR586002
NM10364	Rectum	B2	M	B: P1.17-6,23: F3-36: ST-3200	ERR586009
ST-1976 (cc-)					
NM9071*	Rectum	No	M	B: P1.22-1,14: F5-2: ST-1976	ERR585998
Others					
NM8307	Rectum	No	M	B: P1.12,23: F3-9: ST-5417	ERR585984
NM9124	Rectum	D4	M	Z: P1.22,14-13: F5-7: ST-10866	ERR585999
NM10492	Urethra	No	M	Z: P1.18,25-15: F5-7: ST-3882	ERR586011
NM10763	Urethra	L12	M	Z: P1.22-4,14-13: F5-7: ST-10866	ERR586013
NM8558	Rectum	G7	M	B: P1.12,16: F1-5: ST-897	ERR585988
NM8674	Rectum	H8	M	cnl: P1.18-4,25: F4-1: ST-1136 (cc1136)	ERR585994
NM8602	Rectum	E5	M	cnl: P1.19-2,13-1: F1-62: ST-2153 (cc162)	ERR585991
Invasive meningococcal disease *N. meningitidis* isolates					
cc11					
NM9954*	Joint fluid	Unknown	F	C: P1.5-1,10-8: F4-1: ST-11	ERR586005
LNP27256	Unknown	Unknown	M	C: P1.5-1,10-8: F3-6: ST-11	PRJNA215157
cc23					
NM10313*	Blood	Unknown	M	Y: P1.5-2,10-1: F4-1: ST-10732	ERR586008
cc41/44					
NM8250	Blood	Unknown	F	B: P1.7-2,13-9: F1-25: ST-8052	ERR585983
NM9062	Blood	Unknown	F	B: P1.7-2,4: F1-5: ST-41	ERR585997
NM9565	Joint fluid	Unknown	M	B: P1.12-1,9: F1-5: ST-10698	
NM10864	Blood	Unknown	M	B: P1.7-2,4: F1-5: ST-2314	ERR586017
NM10863	Blood	Unknown	M	B: P1.7-2,4: F3-9: ST-10868	ERR586016
NM11067	CSF	Unknown	M	B: P1.17,16-3: F5-7: ST-136	ERR586019
cc269					
NM10052*	CSF	Unknown	M	B: P1.22,9: F4-1: ST-1161	ERR586006
NM10053*	Blood	Unknown	M	B: P1.22,9: F4-1: ST-1161	ERR586007
cc4821					
M14 240580	Unknown	Unknown	Unknown	B: P1.17-6,23: F3-36: ST-3200	ERR985730
053442	CSF	Unknown	Unknown	C: P1.7-2,14: F3-3: ST-4821	CP000381
ST-1976 (cc-)					
NM9905*	Blood	Unknown	M	B: P1.22-1,14: F5-2: ST-1976	ERR586004

*Isolates containing *aniA* gene with premature stop codon and therefore a putatively non-functional *aniA* gene. All of the remaining isolates contained putatively functional *aniA* genes consistent with these not containing premature stop codons.

A1: concomitant rectal and urethral *N. gonorrhoeae* isolates, both ST-9363, NG MAST 2992 (accession numbers: SAMN04624289 and SAMN04624250).

B2: rectal *N. gonorrhoeae* ST-11516, new NG MAST (SAMN04623631).

C3: concomitant rectal (ST-1584; NG MAST ST-4528, SAMN04624549) and urethral (ST-8122; NG-MAST ST-292, SAMN04621973) *N. gonorrhoeae* isolates, *N. meningitidis* isolates were however unavailable.

D4: urethral *N. gonorrhoeae* (isolate unavailable).

E5: urethral and rectal *N. gonorrhoeae* isolates only one available for analysis ST-7363, NG MAST 2400 (SAMN04624221).

G7: nasopharyngeal *N. gonorrhoeae* ST-7363, NG MAST 2400 (SAMN04624196).

H8: nasopharyngeal *N. gonorrhoeae* ST-1901, NG MAST 1407 (SAMN04624415).

I9: rectal *N. gonorrhoeae* ST-7360, NG MAST 1407 (SAMN04624571).

J10: urethral (ST-11516 NG MAST 1780, SAMN04624544), nasopharyngeal and rectal, (both ST-1901 NG MAST 1407, SAMN04624515 and SAMN04624532) *N. gonorrhoeae.*

K11: nasopharyngeal *N. gonorrhoeae* ST-11463, NG MAST 2992 (SAMN04621963).

L12: rectal *N. gonorrhoeae* ST-7363, NG MAST 10149 (SAMN04623685).

CSF, cerebrospinal fluid.

The 14 *N. gonorrhoeae* isolates corresponded to 2 ST-9363 (NG-MAST ST-2992) isolates (one urethral, one rectal) obtained from one patient on the same day; 5 ST-7363 (NG-MAST ST-2400) or ST-11516 (new NG-MAST) isolates obtained from five individuals between 2011 and 2013; 3 ST-1901 (NG-MAST ST-1407) isolates, 2 of which (pharyngeal and rectal samples) were obtained on the same day from the same patient and a pharyngeal isolate from another patient.

### Antimicrobial resistance

None of the meningococci exhibited phenotypic resistance to fluoroquinolones or macrolides according to the BSAC guidelines (see online supplementary table S1);^16^ however, 15 isolates contained NEIS1753 (*penA*) alleles with known amino acid substitutions associated with resistance to penicillin.^22^ Of these, 14/15 showed reduced susceptibility phenotypically to penicillin (MIC range 0.13–0.5). Three isolates displayed reduced susceptibility to penicillin phenotypically but did not contain a corresponding resistant genotype (MIC range 0.25–0.5).

10.1136/sextrans-2016-052781.supp1 supplementary data

Four of the 14 *N. gonorrhoeae* isolates (29%) were resistant to β-lactams, including third-generation cephalosporins and fluoroquinolones (data not shown). Three isolates without mosaic *penA* alleles exhibited a deletion in the promoter region of the efflux pump regulator, *mtrR*, associated with increased expression of the efflux pump. In the remaining seven isolates, genotypic resistance markers were not detected. Neither mutations in 16S rRNA or 23S rRNA conferring resistance to spectinomycin or azithromycin, respectively, nor plasmid-mediated AMR was detected in any isolate.

### *N. meningitidis* genomic analyses

**Figure 1:**
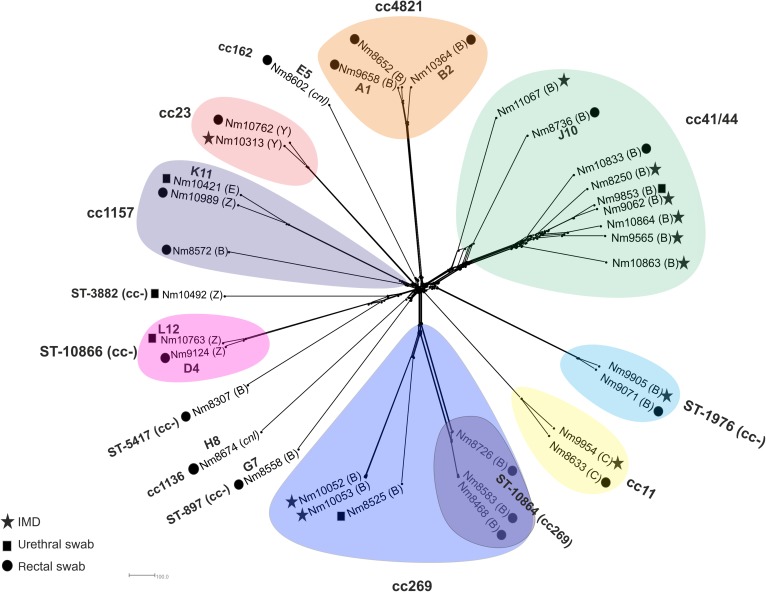
*Neisseria meningitidis* genome comparison. NeighborNet tree depicting *N. meningitidis* isolates compared using 1605 loci core to the meningococcal genome (*N. meningitidis* cgMLST V.1.0). Stars depict invasive meningococcal disease cases; black squares *N. meningitidis* isolates obtained from urethral samples; black circles *N. meningitidis* isolates retrieved from rectal swabs. A1, B2, D4 and so on indicate concomitant *N. gonorrhoeae* infections. (B) serogroup B, (C) serogroup C, (E) serogroup E, (Y) serogroup Y, (Z) serogroup Z, (cnl) capsule null. IMD, invasive meningococcal disease

Genomic comparison of meningococcal core loci were phylogenetically reconstructed with isolates clustering by lineage. Of the eight clusters, five included both invasive and urogenital/rectal isolates (figure 1). Locus differences were used to assess relatedness and possible involvement in transmission networks, with differences in ≤20 loci being considered compatible with recent transmission.^24^ Two ST-1976 isolates (figure 1), NM9071 (rectum) and NM9905 (IMD), showed matching strain designation (B: P1.22-1,14: F5-2) with cgMLST analysis identifying 130 locus differences.

The clonal complex cc41/44 isolates, NM9062 (blood culture) and NM9853 (urethral), (figure 1) exhibited the same strain designation (B: P1.7-2,4: F1-5: ST-41) and contained distinctive alleles not found in the other cc41/44 isolates in this collection. NM8736 was distinct from other cc41/44 strains in this study (see online supplementary figure S1A).

CC269 isolates, NM8468, NM8583 and NM8726, all with the same strain designation (B: P1.19,15-1: F1-5: ST-10864 cc269) and all rectal isolates, formed a distinct cluster (figure 1). NM8468 and NM8583 differed in 4 loci, whereas NM8726 differed from these in a further 108 loci. NM10052 and NM10053 also from cc269, but from IMD, differed in 12 loci. These isolates were distinct from the other cc269 strains. Divergence was apparent in isolates NM9124 (rectum) and NM10763 (urethra) although both of these had the same strain designation (Z:P1.22, 14-23:F5-7:ST-10866) (figure 1). These isolates differed in 122 loci, including loci associated with energy and DNA metabolism, hypothetical proteins and loci implicated in pilin biogenesis.

The French MSM outbreak meningococcus, LNP27256, had the same strain designation as isolate NM8633 (C:P1.5-1,10-8:F3-6:ST-11)^12^ but exhibited 114 locus differences, whereas 313 locus differences were apparent between these and NM9954 (IMD non-MSM). The gene, *aniA*, encoding nitrate reductase and facilitating persistence in anaerobic environments, was found to be functional in all but 8/36 (22%) isolates in this dataset (table 1).^25^

Isolates NM103364, NM8652 and NM9658 belonged to cc4821, which is associated with IMD in China and one case of cc4821 IMD (isolate M14-240580) has been identified in the UK in 2014 (MRF Meningococcus Genome Library http://www.meningitis.org/research/genome). These isolates were distinct from the Chinese isolate, 054332 (see online supplementary figure S1B) and isolates NM10364 and M14-240580 clustered separately from other cc4821 meningococci, consistent with their different PorA types. A total of 362 locus differences were observed between the two clusters with 108 locus differences found between NM10364 and M14-240580.

### *N. gonorrhoeae* genome analyses

**Figure 2:**
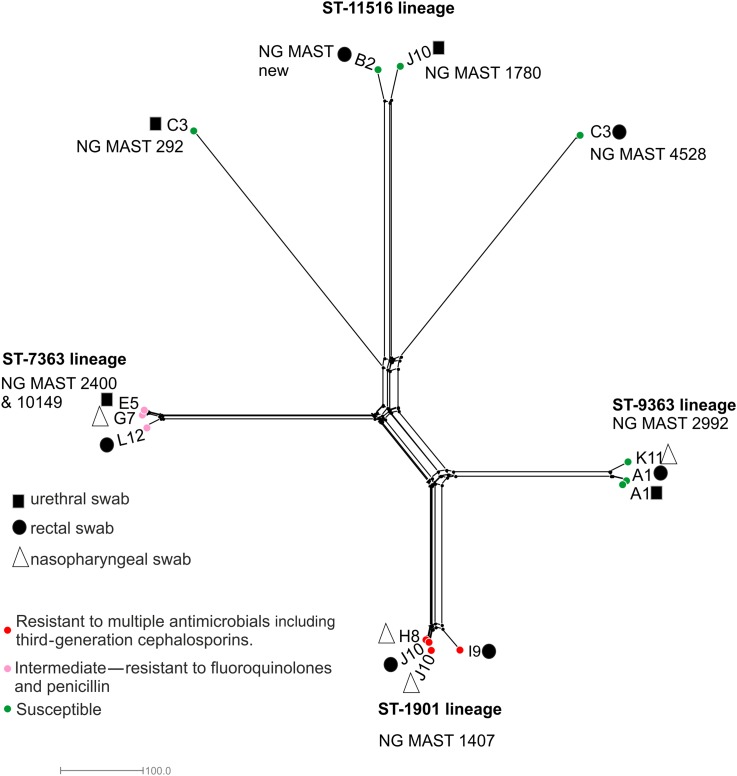
*Neisseria gonorrhoeae* genome comparison. NeighborNet tree depicting *N. gonorrhoeae* isolates compared using the 1668 loci core to the gonococcal genome (*N. gonorrhoeae* cgMLST V.1.0). Black squares depict isolates obtained from urethral samples; black circles isolates retrieved from rectal swabs with triangles indicating nasopharyngeal isolates. Red circles depict gonococci resistant to multiple antimicrobial compounds including third-generation cephalosporins and fluoroquinolones; pink circles indicate gonococci resistant to fluoroquinolones and penicillin, while green circles depict antimicrobial susceptible gonococci.

Core genome analysis identified four distinct clusters (figure 2). These included the ST-1901 genotype, comprising four isolates, one of which ST-7360; the ST-7363 and ST-9363 lineages containing three isolates each and the ST-11516 lineage including two isolates. The remaining two isolates were located on long branches distantly related to ST-11516.

A total of 106 locus differences were observed between isolates in the ST-1901 lineage. This decreased to 25 locus differences after exclusion of the ST-7360 isolate, decreasing to 2 locus differences (15 polymorphic sites due to an indel in a hypothetical protein) once the 2 ST-1901 isolates from patient J10 were compared alone. Patient J10 also had a urethral *N. gonorrhoeae* infection; however, this was due to a genomically distinct ST-11516 isolate (figure 2). A total of 72 locus differences were observed between isolates in the ST-7363 cluster. This decreased to 22 locus differences when isolates from patients E5 and G7 only were compared. One variant locus, due to a frame-shift in a homopolymeric tract of the gene *hpuA* implicated in iron acquisition from haemoglobin–haptoglobin, was observed between gonococci from patient A1 (rectum and urethra).

## Discussion

Urogenital and rectal meningococcal infections have been increasingly reported since the 1930s,^26^ with recent accounts associating meningococci expressing serogroup C capsules with IMD outbreaks among MSM.^8^ ^9^ In this study, the majority of urogenital and rectal meningococci analysed belonged to hyperinvasive lineages (table 1, figure 1). This contrasts with the situation in nasopharyngeal carriage, where the prevalence of hyperinvasive lineages is lower.^27^ Meningococcal carriage is dominated by less invasive meningococci, which are frequently acapsulate or express capsules not associated with IMD. This suggests that common genetic determinants may be involved in promoting both urethral/rectal colonisation and IMD. As the rectum and urethra are physiologically distinct from the nasopharynx, factors such as the polysaccharide capsule, a well-known meningococcal virulence determinant, may be important for colonisation and persistence (table 1), consistent with the prevalence of encapsulated strains identified here and previously.^28–30^

The expansion of meningococci into this environment may result in adaptation leading to the emergence of strains that are able to persist by novel means of colonisation and transmission. An example is the gene *aniA* encoding nitrite reductase and essential for the growth of gonococci under oxygen-limiting conditions. Both urogenital and invasive cc11 *N. meningitidis* isolates from MSM have been found to express this gene as opposed to invasive strains obtained from non-MSM cases, which do not.^25^ Gene-by-gene comparisons undertaken here between MSM and non-MSM derived cc11 meningococci identified further variation in genes implicated in the respiratory chain as well as Na^+^ translocating NADH-ubiquinone reductase subunits, which are essential for ATP synthesis. These changes are consistent with adapative metabolic changes that may promote adaptation. Further comparisons between pairs of invasive and urogenital/rectal meningococci from the same lineage, identified an average of 125 locus differences, comprising genes associated with core metabolic functions such as amino acid biosynthesis, DNA metabolism and central intermediary metabolism. Such changes may promote adaptation to the urogenital tract.

Until now, there has been relatively little interest in the population dynamics of urogenital/rectal meningococci. Our findings, however, indicate that investigation of such strains is warranted, particularly as urogenital and rectal carriage may promote the introduction and transmission of potentially invasive meningococci. For example, both of the ST-1976 strains described shared the same PorA, FetA genes with only nine other isolates with this ST recorded in the PubMLST database at the time of writing, none of which from the UK. We also identified three urogenital/rectal serogroup B cc4821 meningococci (table 1). IMD outbreaks due to cc4821 meningococci were previously limited to Asia, particularly China; however, a case of IMD due to cc4821 was identified for the first time in the UK in 2014 {MRF Meningococcus Genome Library http://www.meningitis.org/research/genomehttp://www.meningitis.org/research/genome, #7406}. The strain designation of this isolate was identical to the cc4821 rectal strains, but distinct from Chinese cc4821 meningococci (see online supplementary figure S1B).^31^ The continued presence of cc4821 in the UK indicates that it should be monitored, since coverage with the serogroup B vaccines, Bexsero and Trumenba, has not been determined for strains from this clonal complex.^32^

As potential pathogens, urogenital and rectal meningococci pose a risk to public health. Their frequent coexistence with gonococci could promote the transfer of AMR genes (figure 2). This is particularly important in gonococci exhibiting decreased susceptibility to third-generation cephalosporins mediated through recombination of mosaic *penA* alleles. The annotation of genes across the *Neisseria* genus enabled by pubMLST.org/neisseria uniquely allows identification of horizontal genetic transfer events between species. We found evidence of such events; for example, *N. meningitidis* isolate NM8633 possessed a *penA* allele more commonly found in gonococci (see online supplementary table S1), although the *penA* gene did not contain motifs associated with resistance. Gonococci from two of our cases were resistant to multiple antimicrobials. These possessed the gonococcal genetic island, a type IV secretion system known to secrete single-stranded DNA and promote recombination.^33^ It is therefore possible that acquisition of resistance genes by meningococci could be enhanced in such situations; indeed, urogenital meningococci possessing gonococcal plasmids have been described.^34^

The prevalence of colonisation with meningococci is likely to be underestimated, as routine methods are optimised to recover gonococci rather than meningococci from sexual health samples. Furthermore, pharyngeal meningococci were not available for comparison. Nevertheless, data in this study show that urogenital/rectal meningococci are diverse and that the majority of these were encapsulated and belonged to hyperinvasive lineages. Urogenital/rectal meningococci are worthy of more attention as sexual transmission may lead to the dispersal of potentially invasive strains.

**Key messages**

- Urogenital/rectal colonisation by *Neisseria meningitidis* is associated with encapsulated members of hyperinvasive lineages, which contrasts with nasopharyngeal carriage where the prevalence of such meningococci is low.
- Although urogenital/rectal carriage with *N. meningitidis* may be asymptomatic, colonising organisms have the capacity for invasion.
- Co-colonisation of potentially invasive meningococci with multidrug-resistant gonococci may promote the transfer of antimicrobial resistance.
