# Supplementary material for: Genomic analysis of urogenital and rectal Neisseria meningitidis isolates reveals encapsulated hyperinvasive meningococci and coincident multidrug-resistant gonococci
Source: Sex Transm Infect. 2017 Jan 30;93(6):445–51. doi: 10.1136/sextrans-2016-052781 (PMC5574384; doi:10.1136/sextrans-2016-052781)
Supplement: supplementary data [file sextrans-2016-052781supp001.pdf]

Table S1. Allelic profiles of loci associated with antimicrobial resistance (NEIS loci) and AMR phenotype (P).

	Beta-lactams					Fluoroquinolones				Macrolides				Plasmids		Efflux pump	
	**NEIS0408 (*pilQ*)**	**NEIS0414 (*ponA*)**	**NEIS1753 (*penA*)**	**NEIS2020 (*porB*)**	**P**	**NEIS1320 (*gyrA*)**	**NEIS1525 (*parC*)**	**NEIS1600 (*parE*)**	**P**	**16S rRNA**	**23S rRNA**	**NEIS0149 (*rpsE*)**	**P**	**NEIS2210 (*tetM*)**	**NEIS2357 (*blaTEM*)**	**NEIS1635 (*mtrR*)**	***pro*NEIS1635 (*mtrR* promoter)**
NM8250	71	130	5	27	S	9	10	25	S	*	*	1	S	0	0	37	9
NM8307	277	296	**371**	1182	R	287	309	206	S	1542	522	1	S	0	0	7	5
NM8468	455	2	**373**	1183	I	302	311	338	S	*	*	1	S	0	0	247	5
NM8525	70	62	62	199	S	202	19	54	S	*	54	45	S	0	0	10	7
NM8558[a]	456	28	**375**	1184	R	303	704	339	S	1543	523	1	S	0	0	15	6
NM8572	4	11	**354**	42	R	249	31	7	S	*	524	1	S	0	0	OFF	5
NM8583	455	2	**373**	1183	R	302	311	338	S	*	*	1	S	0	0	247	5
NM8602[a]	33	28	33	149	S	35	313	56	S	1384	525	1	S	0	0	251	5
NM8633	116	1	377[b]	1	S	1	1	1	S	*	1	1	S	0	0	2	5
NM8652	359	7	**380**	265	R	255	275	288	S	*	526	1	S	0	0	211	5
NM8674[a]	241	28	**112**	1185	I	32	30	60	S	1412	112	1	S	0	0	7	5
NM8726	455	2	**373**	1183	I	302	311	338	S	*	*	1	S	0	0	247	5
NM8736[a]	16	16	5	45	S	32	8	25	S	1341	1	0	S	0	0	273	5
NM9062	87	2	5	260	S	9	135	25	S	1407	424	1	S	0	0	107	5
NM9071	347	1	41	38	I	31	26	280	S	1324	528	1	S	0	0	274	5
NM9124[a]	277	61	382	477	R	7	314	341	S	1544	*	1	S	0	0	275	5
NM9565	16	129	**112**	42	R	248	17	25	S	1310	20	1	S	0	0	9	5
NM9658[a]	359	7	**380**	1186	R	255	275	288	S	1	*	1	S	0	0	211	5
NM9853	457	2	5	1187	S	9	135	25	S	1310	20	1	S	0	0	9	5
NM9905	347	1	41	38	S	31	26	280	S	1324	528	1	S	0	0	7	5
NM9954	16	1	3	1	S	1	1	1		*	1	1	S	0	0	8	5
NM10052	99	68	88	198	S	10	29	54	S	1379	191	45	S	0	0	59	5
NM10053	99	68	88	198	I	10	29	54	S	1379	191	45	S	0	0	59	5
NM10313	109	45	8	67	S	107	70	79		1332	529	1	S	0	0	7	5
NM10364[a]	458	78	**319**	126	R	255	275	288	S	*	526	1	S	0	0	211	5
NM10421[a]	4	64	38	477	S	304	47	58	S	1386	197	1	S	0	0	35	5
NM10492	109	297	385	1188	R	305	309	348	S	1545	530	1	S	0	0	57	10
NM10762	109	45	8	262	S	107	70	79	S	1332	268	1	S	0	0	7	5
NM10763[a]	277	298	382	1189	R	7	314	341	S	1544	*	1	S	0	0	275	5
NM10833	459	45	**352**	234	R	9	17	25	S	*	20	1	S	0	0	8	5
NM10853	3	6	**109**	27	S	20	13	19		1546	531	1	S	0	0	41	5

NM10863	7	2	386	45	S	9	84	25		1547	20	1	S	0	0	276	5
NM10864	16	129	**112**	42	I	9	17	25	S	1310	20	1	S	0	0	9	5
NM10989	4	64	**388**	43	I	79	47	58	S	1548	197	1	S	0	0	8	5
NM11067	460	19	5	1063	S	32	8	36	S	1344	20	1	S	0	0	9	5

a: dual *N. meningitidis/N. gonorrhoeae* infections; b: *penA* allele more commonly found in *N. gonorrhoeae*; * incomplete locus; OFF premature stop codon in locus putatively resulting in loss of function; bold alleles denote the presence of known mutations associated with conferring resistance as described previously [1].

P denotes phenotypic AMR deduced from MIC values with S: susceptible (MIC ≤ 0.06), I: intermediate (MIC 0.007 – 0.24), R: resistant (MIC ≥ 0.25).

Figure S1. Further genomic analyses of cc41/44 and cc4821 *N. meningitidis* isolates

A.

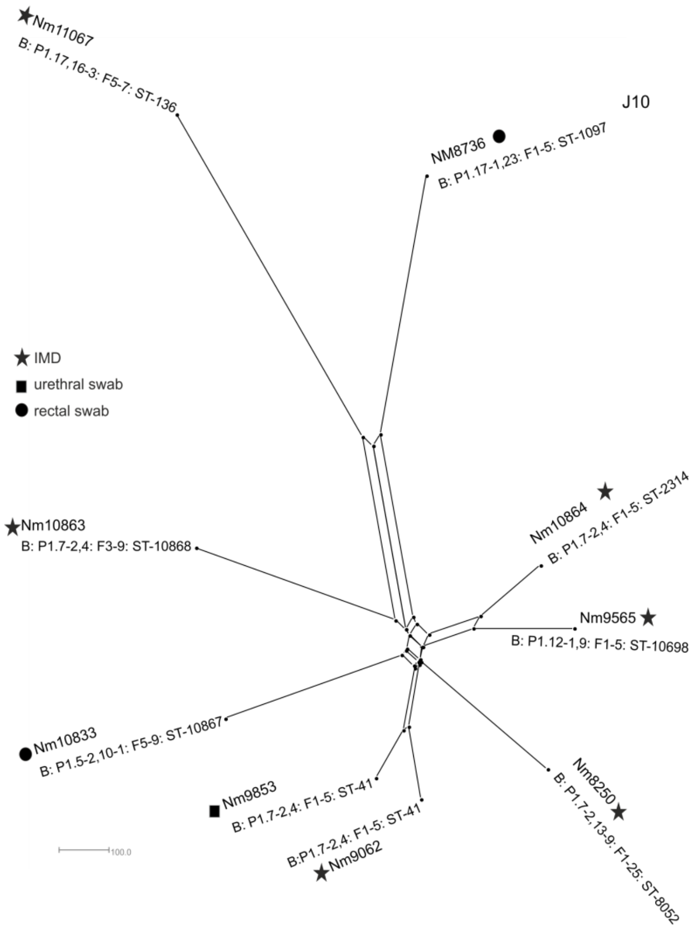

B.

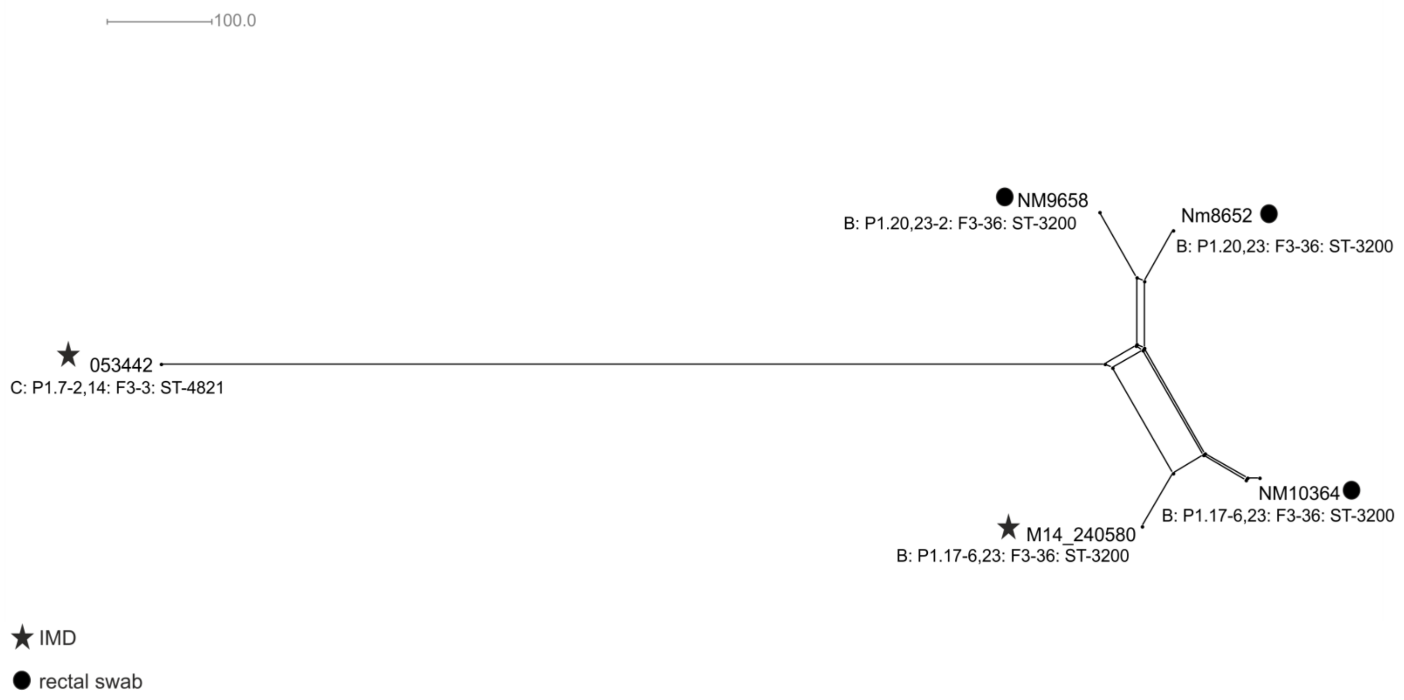
100.0
NM9658
B: P1.20,23-2: F3-36: ST-3200
Nm8652
B: P1.20,23: F3-36: ST-3200
053442
C: P1.7-2,14: F3-3: ST-4821
NM10364
B: P1.17-6,23: F3-36: ST-3200
M14_240580
B: P1.17-6,23: F3-36: ST-3200
IMD
rectal swab
